# Effect of different dentin etching protocols on the immediate microtensile bond strength of two bulk-fill restorative systems in class I cavities

**DOI:** 10.1186/s12903-026-07926-2

**Published:** 2026-03-11

**Authors:** Eman H. Albelasy, Ahmed Gamal Raghip, Hoda Saleh Ismail

**Affiliations:** 1 Faculty of Dentistry, Conservative Dentistry Department, Mansoura University, 1 Algomhoriya St, Mansoura, 35511 Egypt; 2 Faculty of Oral and Dental Medicine, Restorative Dentistry Department, Alsalam University, Tanta, Egypt

**Keywords:** Microtensile bond strength, Self-cure, C-factor, Short-dentin etching

## Abstract

**Background:**

Reliable bonding to dentin in high C-factor cavities remains a clinical challenge due to polymerization stress. Bulk-fill composites, including self- and dual-cured systems, aim to simplify placement and improve adaptation in deep cavities. However, limited data exist on how different dentin surface treatments affect their bond strength.

**Materials and methods:**

Sixty extracted human molars with standardized Class I cavities were randomly assigned to six groups based on restorative system (self-curing bulk-fill, and a dual-cured bioactive composite system) and dentin treatment (self-etch, short 3-second etch, conventional etch-and-rinse). µTBS was evaluated after one month of water storage. Two-way ANOVA was used to assess the effects of material and dentin treatment, and failure modes were analyzed descriptively using Chi-square test.

**Results:**

Dentin treatment significantly affected µTBS (*p* < 0.001), whereas the restorative system did not (*p* = 0.56). The highest µTBS was observed in the etch-and-rinse groups for both the self-cure (26.4 MPa) and the dual-cure systems (26.0 MPa), while the lowest values occurred in self-etch groups (self-cure: 18.0 MPa; dual-cure: 19.5 MPa). Failure mode analysis showed predominantly adhesive failures in self-etch groups and mixed failures in etch-and-rinse groups.

**Conclusion:**

Within the limitations of this study, etch-and-rinse dentin treatment appears to provide higher µTBS than self-etch. While short dentin etching could potentially offer a practical compromise, particularly for the self-curing bulk-fill composite.

**Clinical significance:**

A short 3-second etch may improve bond strength over self-etch protocols, especially for the self-cure restorative system (Stela, SDI), suggesting a simple strategy to optimize adhesion while minimizing potential over-etching.

## Background

The field of adhesive dentistry has become a cornerstone of modern dental practice following Dr. Buonocore’s seminal work on acid etching of enamel [1]. This innovation laid the foundation for bonding techniques that have since revolutionized restorative procedures. With the decline in the use of dental amalgam and a growing emphasis on minimally invasive operative dentistry, resin composites have gained prominence as the material of choice for direct restorations [2]. However, achieving reliable adhesion in dentin remains more challenging than in enamel, primarily due to dentin’s lower inorganic content and higher water content, which complicate bonding procedures [3]. Dentin is a complex biocomposite tissue that has been characterized either as a heterogeneous assembly of different dentin types or as a bone-like nanocomposite consisting of carbonated hydroxyapatite, proteins, and water [4]. Unlike enamel, dentin is a biologically dynamic and heterogeneous substrate, which makes adhesive bonding less predictable and highly sensitive to both biological and clinical factors, including dentin depth, permeability, pulpal pressure, and substrate condition [5, 6]. 

As a result, bonding agents and restorative materials have undergone substantial advancements to overcome these challenges [7]. A major objective in adhesive dentistry today is the simplification of bonding protocols while enhancing the longevity and performance of resin-dentin interfaces. These developments have simplified clinical application protocols [8], exemplified by the shift from multi-step etch-and-rinse (ER) systems to universal one-bottle adhesives [7]. 

Notably, universal or multi-mode adhesives have become increasingly popular for their adaptability and versatility in bonding to a wide range of dental substrates [9]. Despite their clinical appeal, universal adhesives have sparked ongoing debate regarding the most effective method of application, particularly in relation to the dentin substrate [10–14]. Although earlier studies supported the use of universal adhesives in self-etch mode for sustained in vitro performance [15], more recent in vivo investigations have produced inconsistent findings [16, 17]. A growing body of evidence indicates that the performance of universal adhesives is influenced by how they are applied [10]. A new approach to enhance resin–dentin bonding, known as short dentin etching (SDE), has been introduced. This method involves applying phosphoric acid to the dentin surface for 3 s, followed by rinsing and drying, resulting in a moderately demineralized substrate [18]. This approach appears promising for increasing the bond strength of simplified adhesives, as indicated by earlier studies [11, 18–20]. Nonetheless, additional investigation is required to confirm its reliability.

In addition to the simplifications of the adhesive system, bulk fill composites were developed to improve the curing depth, allowing restorations to be placed in larger increments or in bulk, thus saving clinical chair-time [21]. Despite these advancements, certain challenges remain most notably, polymerization shrinkage during light-curing procedures [22, 23]. 

This volumetric contraction can compromise the marginal seal between resin composites and dental hard tissues like dentin and enamel [12, 24]. Furthermore, incremental placement of direct resin composites may lead to the incorporation of voids, which can compromise the mechanical strength of the restoration [25]. 

To address this, a novel self-curing restorative system (Stela, SDI Ltd, Australia) has recently entered the market. Unlike traditional light-cured materials, Stela is a bulk-fill, self-curing resin-based composite [26], used in conjunction with a special adhesive primer. This primer does not require light activation, initiating polymerization upon contact with the restorative material [27]. Self-cured composites have been reintroduced as a direct restorative option because they generally exhibit lower polymerization shrinkage stress, attributable to reduced volumetric shrinkage, a prolonged pre-gel phase, and slower polymerization kinetics, as well as an unlimited depth of cure [28, 29]. Similarly, Activa Bioactive Restorative (Pulpdent Corporation, Watertown, MA, USA) is marketed as a dual-cure restorative material with low volumetric polymerization shrinkage (approximately 1.7%) and the ability to be placed in 4–5 mm increments, which may contribute to reduced chair time and lower technique sensitivity [30, 31]. ACTIVA BioACTIVE Restorative (Pulpdent) is marketed as an “ionic resin” combining a resin-composite matrix with a polyacid-modified, glass–ionomer–like phase [32]. Laboratory and manufacturer data suggest that its reactive glass fillers release fluoride and calcium, while the resin phase provides light-curing and improved flexural properties [32]. 

Randomized controlled trials involving adults and adolescents have demonstrated comparable short- to medium-term clinical performance of ACTIVA relative to nanohybrid and bulk-fill resin composites, with similar incidences of marginal discoloration, postoperative sensitivity, and secondary caries over follow-up periods of up to two years [33–35]. Accordingly, both materials were selected in this study to represent contemporary bulk-fill restorative systems with reduced polymerization stress but distinct curing mechanisms, allowing evaluation of whether short dentin etching can produce stable dentin bonding and marginal integrity across materials with different polymerization kinetics and bonding strategies.

Effective bonding is crucial for reducing adverse outcomes such as post-operative sensitivity, marginal staining, secondary caries, and harmful effects on the dental pulp [36]. In general, laboratory bond strength testing is conducted to evaluate the dentin adhesion performance of newly developed restorative materials compared to earlier versions [37]. Among these methods, microtensile bond strength (µTBS) testing is one of the most commonly used techniques, offering several advantages over traditional bond strength tests, such as the ability to assess interfacial bond strength on very small areas, often less than 1 mm² [38, 39]. However, it is technique-sensitive, labour-intensive, and associated with potential specimen damage and dehydration [40]. 

While most studies on dentin bond strength have been conducted using flat bonding surfaces, limited data are available on how varying C-factors influence the potentially detrimental effects of cavity geometry on dentin bond strength [41–43]. Therefore, this study aimed to evaluate and compare the micro tensile bond strength of a self-cure and a dual-cure resin composite to dentin following different surface pretreatment protocols (no etch, short dentin etching, and 15-sec etching). The null hypotheses were that (1) there is no difference in bond strength between the two restorative systems regardless of etching protocol; (2) etching duration has no effect on bond strength within each restorative system.

## Materials and methods 

### Sample size calculation

In the absence of previously published data suitable for powering a two-factor design, the calculation was based on the most clinically relevant main-effect comparison reported in a previous study [18]. That study reported mean µTBS values of 31.21 ± 6.87 MPa and 42.97 ± 7.12 MPa for the self-etch and short-etching protocols, respectively, corresponding to an effect size of Cohen’s f = 0.84. Using a significance level (α) of 0.05 and a statistical power (1 − β) of 0.80, the minimum required sample size was estimated to be 30 teeth. Because the present study involved a two-factor experimental design with six groups, the sample size was increased to 10 teeth per group. The tooth was considered the statistical unit. It is acknowledged that basing the calculation on a one-way ANOVA model may not fully account for potential interaction effects, which represents a limitation of this approach.

### Sample preparation 

Table 1 outlines the specifics of the materials, components, manufacturers, and batch numbers for the materials utilized in this investigation. A total of 60 sound, fully erupted, non-carious human molars were used. The teeth were extracted for periodontal reasons and obtained from healthy individuals undergoing dental treatment at the Oral and Maxillofacial Surgery Department Clinic, Faculty of Dentistry, Alsalam University. All procedures conducted were in accordance with the Helsinki Declaration and a protocol approved by the institution’s ethical review board (SUE010607251). Informed consent was obtained from all patients, who were notified that their extracted teeth would be used for research purposes. Following extraction, the teeth were disinfected in a 0.5% chloramine solution, then cleaned using rubber cups and a fluoride-free pumice paste. They were subsequently stored in distilled water within an incubator for no longer than one month before use. Each tooth was embedded in self-curing acrylic resin, with the resin level positioned 2 mm below the cementoenamel junction (CEJ) to facilitate handling. All teeth were sectioned parallel to the occlusal surface and perpendicular to the long axis using a low-speed diamond saw (IsoMet 4000, Buehler Ltd., Lake Bluff, IL, USA) under continuous water cooling.

The occlusal enamel was removed using a low-speed, water-cooled diamond saw (IsoMetTM 4000, Buehler Ltd., Lake Bluff, IL, USA) to expose a flat dentin surface. An experienced operator (9 years) visually examined the prepared surfaces under 5× magnification using loupes (Univet, Italy) to confirm the absence of residual enamel. Surfaces were then treated with 30–40% phosphoric acid for 3–5 s to verify complete enamel removal, followed by the removal of an additional 0.1 mm of surface material [44]. 

Standardized Class I cavities (3 mm × 3 mm × 2 mm) were prepared using a straight carbide bur in a high-speed handpiece (Sirona T4, Bensheim, Germany) under continuous air-water cooling. The bur was replaced after every five preparations. The cavity floors and axial walls were smoothed using a small piece of 600-grit silicon carbide paper in a circular motion with continuous water flow for 60 s to standardize the smear layer [44, 45]. Digital periapical radiographs were obtained following cavity preparation to verify adequate remaining dentin thickness. A remaining dentin thickness (RDT) of ≥ 1.0 mm between the cavity floor and the pulp chamber was considered acceptable [46]. Teeth with pulp exposure were excluded from the study.

### Experimental design and restorative procedures

The 60 teeth were randomly allocated using a computer-generated random sequence into two groups (*n* = 30 each) based on the type of restorative material used: Stela (SDI Ltd, Australia) and ACTIVA Bulk-Fill Flow (Pulpdent Corporation, Watertown, MA, USA). Each of these main groups was further subdivided into three subgroups (*n* = 10) according to the dentin conditioning method employed: self-etch (SE) using Stela primer and universal adhesive (G-Premio Bond with ACTIVA), short dentin etching (SDE) for 3 s with 37% phosphoric acid, and etch-and-rinse (ER) (15 s). Materials were applied according to manufacturers’ instructions, with Stela used alongside its proprietary primer, and ACTIVA applied with a universal adhesive (G-premio Bond, GC Corporation, Tokyo, Japan). In the self -etch groups (SE), the adhesives were brushed into the cavities using a microbrush for 20 s, followed by 5 s of air drying to evaporate the solvent. In the ER groups, dentin was etched with a 37% orthophosphoric acid gel for 15 s, followed by rinsing with distilled water for 15 s. The dentin surface was then air-dried for 10 s using an oil-free three-way syringe, held at a 45° angle and approximately 1.5 cm from the target area, with the air pressure regulated at 1 bar [19, 45, 47]. For the short-dentin etching protocol, phosphoric acid was applied for 3 s, rinsed with distilled water for 15 s, and the dentin was air-dried in the same manner as the 15-second etch to maintain a slightly moist surface.

**Fig. 1:**
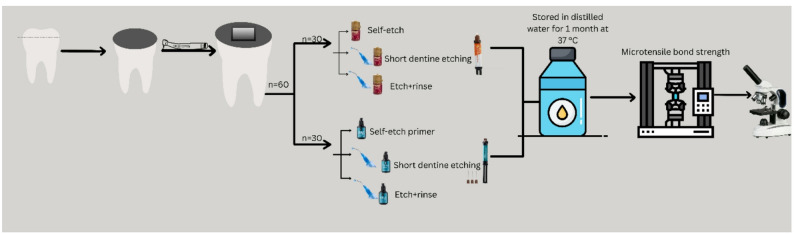
Schematic overview of the experimental workflow. Sixty extracted human molars were prepared to expose mid-coronal dentin, and standardized Class I cavities were created. The specimens were randomly divided into two main groups according to the restorative system used (self-cure bulk-fill and dual-cure bulk-fill; *n* = 30 each). Each group was further subdivided based on the bonding strategy employed: self-etch (SE), short dentin etching (SDE; phosphoric acid applied for X s), and etch-and-rinse (ER) (*n* = 10 per subgroup). Composite buildups were then fabricated and sectioned to obtain micro-tensile test specimens. All samples were stored in distilled water at 37 °C for one month before micro-tensile bond strength (µTBS) testing. The fractured specimens were scanned with stereomicroscope to determine the mode of failure

All restorative materials and adhesive systems were used according to the manufacturer’s instructions and light-cured (when necessary), through a light-emitting diode curing unit (Elipar Deep Cure, 3 M ESPE, St. Paul, MN, USA) operating at 1000 mW/cm2, which was checked using a radiometer. The specimens were finally restored with the test restorative materials as previously mentioned. The self-curing bulk-fill restorative system (STELA) was placed in a single increment and allowed to self-cure at room temperature for 4 min. For Activa, the material was injected to fill the standardized cavity in a single increment, allowed to self-cure for 10 s, and then light-cured for 10 s with a light-emitting diode curing unit (Elipar Deep Cure, 3 M ESPE, St. Paul, MN, USA) operating at 1000 mW/cm2. A schematic illustration of the experimental protocol is illustrated in Fig. 1.

After all restorative procedures had been completed, all specimens were individually stored in sealed containers filled with distilled water at 37 °C in an incubator for one month. The storage medium was renewed weekly to prevent contamination and maintain consistent conditions.

After the storage period, the teeth were sectioned perpendicular to the adhesive interface using a water-cooled diamond saw (IsoMetTM 4000, Buehler Ltd., Lake Bluff, IL, USA) to produce rectangular micro-specimens (1 mm × 1 mm) for microtensile bond strength (TBS) testing. These specimens were kept hydrated until testing.

Four intact beams per sample were selected from the central portion, avoiding any cracks. Each beam contained both dentin and the bonded composite interface [44]. 

Each beam was attached to a testing jig using a cyanoacrylate adhesive (Zapit, DVA Inc., Corona, CA, USA) and mounted on an Instron Universal Testing Machine (Model 3345, Norwood, MA, USA). A tensile force was applied at a constant crosshead speed of 0.5 mm/min using a 500 N load cell until failure occurred. The tensile bond strength (TBS) was calculated in megapascals (MPa) using Bluehill Lite software (Instron, Norwood), by dividing the force at fracture (in newtons) by the bonded surface area (in mm²). Any stick that failed before testing (pre-testing failure, PTF) was assigned a bond strength of zero and included in the statistical evaluation. Failure modes were examined under a stereomicroscope (MSA 166305) at 50× magnification and classified as cohesive in dentin, adhesive at the interface, cohesive in composite, or mixed. Mixed failures were defined as those with more than 10% of the fractured surface showing both adhesive and cohesive features in either dentin or composite.

### Statistical analysis

The bond strength (MPa) was calculated by averaging the microtensile bond strength (µTBS) values of four beams taken from each tooth. Statistical analysis was carried out using SPSS software (version 20). Normality of the data was confirmed using the Shapiro–Wilk test (*p* > 0.05). Homogeneity of variance was assessed using Levene’s test. Parametric tests were used as both assumptions were reasonably satisfied.

A two-way ANOVA was used to assess the effects of the material type and adhesive strategy on bond strength, followed by an LSD post-hoc test for pairwise comparisons. The distribution of failure modes was analyzed using cross-tabulation and the Chi-Square test.

**Table 1:** Materials used in the study

Material	Manufacturer	Composition	Lot Number	Specification
Stela Primer	SDI, Victoria, Australia)	Methyl ethyl ketone (10–30 %), 4-methacryloxyethyl trimellitic anhydride (10–30 %), acrylic monomer (10–30 %), 10-methacryloyloxydecyl dihydrogen phosphate (10- MDP; 10–30%) and diurethane dimethacrylate (DUDMA; 10–30%) (**)	1,238,390	A self-cure primer
Stela automix	SDI Ltd., Australia	Organic matrix (***): DUDMA (10–25%), glycerol dimethacrylate (GDMA; 5–10 %), ytterbium fluoride (3–7 %) and 10-MDP (1–5%). Filler content (****): Fluoro- alumino-silicate glass: mean particle size 4.0 μm (distribution range approx. 2 to 8 μm) and Barium-alumino- borosilicate glass: mean particle size 2.8 μm (distribution range approx. 2 to 5 μm). Filler loading: 61.2 wt% (36.4 vol%)	1,238,762	Self-cure bulk-fill composite
ACTIVA BIOACTIVE Bulk-flow	Pulpdent Corporation, Watertown, MA, USA	Blend of diurethane and other methacrylates with modified polyacrylic acid MCP resin-modified calcium phosphate Amorphous silica Sodium fluoride	240,806	Bioactive resin-based restorative material with ion-releasing/glass-ionomer–like features
G-Premio Bond	GC Corporation, Tokyo, Japan	10- ethacryloyloxydecyl dihydrogen phosphate, 4-methacryloxyethyl trimellitate, methacryloyloxyalkyl thiophosphate methylmethacrylate, methacrylate monomer, acetone, water, silica, initiator.	2,312,021	Universal adhesive

## Results

### µTBS results

**Fig. 2:**
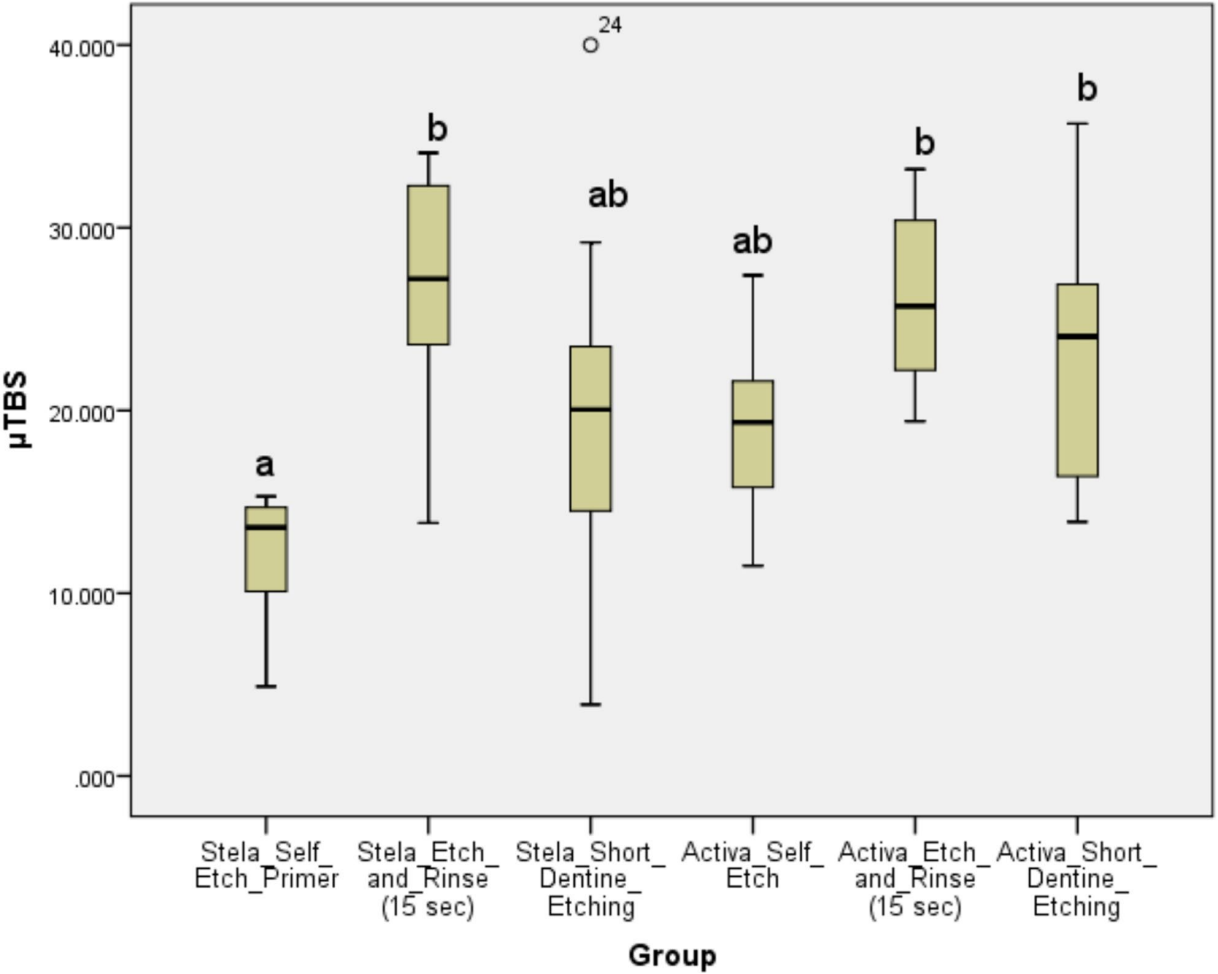
Box plot representing the microtensile bond strength (µTBS) values (in MPa) for the tested groups using different adhesive strategies. Groups labeled with the same letter are not statistically significant(*p* > 0.05)

Table 2, and Fig. 2 present the mean µTBS values and standard deviations for all tested subgroups. Two-way ANOVA showed that the type of restorative material did not significantly affect bond strength values (*p* = 0.56), while the dentin treatment method had a significant influence (*p* < 0.001). No significant interaction was found between the two variables (*p* = 0.238). Both Stela and ACTIVA achieved their highest bond strength values with the etch-and-rinse technique (26.4 MPa and 26.0 MPa, respectively), with no statistically significant difference between the two materials under this protocol (*p* > 0.05). Under self-etch conditions, ACTIVA demonstrated higher bond strength than Stela (19.0 MPa vs. 12.2 MPa), but this difference was not statistically significant (*p* > 0.05). The short dentin etching protocol resulted in intermediate bond strengths for both materials, with ACTIVA showing slightly better performance than Stela; however, the difference was also not statistically significant (*p* > 0.05).

**Table 2:** Mean ± SD (95% confidence interval lower bound-upper bound) of microtensile bond strength values in MPa among tested restorative materials and across the different adhesive strategies

Material	Self-etch mode	Short-dentin etching	Etch-and rinse-
Stela	12.2 ± 3.5^a^ (8.03, 16.36)	19.6 ± 3.9^ab^ (15.4, 23.73	26.4 ± 6.5^b^ (22.2, 30.52)
ACTIVA	19 ± 4.8^ab^ (14.8, 23.16)	23.1 ± 6.8^b^ (18.88, 27.21)	26 ± 4.7^b^ (21.87, 30.2

Groups identified with the same superscripted lower-case letters are not significantly different from each other. (p < 0.05)

### Failure pattern distribution

Table 3 illustrates the distribution of failure modes across all groups presented in percentages. A notable interaction was observed between failure patterns and composite type (*p* = 0.000), and type of dentin surface treatment (*p*=0.000). Across all strategies, Stela generally showed higher mixed failure rates, particularly with short dentin etching (80%) and etch-and-rinse (62.5%). Activa, however, had a higher propensity for adhesive failures in dentin, especially in the self-etch group (77.5%), indicating weaker dentin bonding. Short dentin etching resulted in the most distinct contrast between materials, with Stela favoring mixed failures and Activa showing a broader failure distribution.

**Table 3:** Failure modes distribution among the tested restorative systems and across different adhesive strategies in percentages (number of beams)

Dentin Treatment	Material									
	Stela					Activa				
	Adhesive in dentin	Cohesive in composite	Mixed	Cohesive in dentin	Pre-test failure	Adhesive in dentin	Cohesive in composite	Mixed	Cohesive in dentin	Pre-test failure
Self-etch	40% (16)	0	27.5%(11)	32.5%(13)	7 % (3)	77.5 (31)	7.5(3)	15 % (6)	0	11.1% (5)
Short dentin etching	20% (8)	0	80%(32	0	4.8 % (2)	42.5 (17)	22.5 (9)	15% (6)	20% (8)	7% (3)
Etch-and-rinse	20% (8)	17.5% (7)	62.5 %(25)	0	7 % (3)	25% (10)	25% (10)	40 (16)	10% (4)	9.1 %(4)

## Discussion

This study evaluated the bond strength of two bulk-fill resin composites, one self-cured and one dual-cured, to dentin at the base of Class I cavities. A high C-factor cavity configuration was selected to replicate clinical conditions typically encountered with bulk-fill composites, aiming to assess the materials under challenging but clinically relevant circumstances [48]. Based on the findings, the null hypothesis that there would be no difference in immediate bond strength between the tested restorative systems, a self-curing bulk-fill and a dual-cured bioactive restorative systems, when applied using self-etch, short dentin etching, or etch-and-rinse protocols, was rejected.

Overall, the etch-and-rinse technique consistently produced the highest bond strength values for both materials, while the self-etch mode resulted in the lowest values. Intermediate values were observed with short dentin etching. For the self-cured composite Stela, the etching protocol had a significant impact on bond strength. The self-etch application resulted in the lowest bond strength (12.2 ± 3.5 MPa), which was significantly lower than both short dentin etching (19.6 ± 3.9 MPa) and etch-and-rinse (26.4 ± 6.5 MPa) protocols. For universal adhesives, it is generally advised to selectively etch the enamel, promoting micromechanical retention [49], while relying on the chemical bonding capability of 10-MDP to adhere to dentin [50]. Nonetheless, this study found that bond strength values remained consistently lower when the self-etch (SE) mode was used for both materials, in agreement with previous research underscoring the limitations of SE strategies in achieving strong bonds within high C-factor cavities [51, 52]. Although the morphology of the smear layer has little effect on the bonding effectiveness of adhesives in the etch-and-rinse mode, mild self-etch adhesives may inadequately encapsulate the smear layer, potentially compromising bond quality [53]. Therefore, the etching capacity of self-etch adhesives is strongly related to the adhesive interaction depth in dentin [53]. The primary challenge of the self-etch bonding mechanism is achieving effective smear layer dissolution without causing extensive dentin demineralization, in order to preserve the tightly bound hydroxyapatite crystals at the bonding interface [18]. The Stela primer has a composition comparable to that of HEMA-free universal adhesives, notably containing the functional monomer 10-MDP and being free from both BPA and HEMA. These similarities suggest that Stela may respond to adhesive strategies in a manner comparable to HEMA-free, universal adhesives. Supporting this, previous research [19] reported that the adhesive strategy had a significant impact on the dentin bond strength of such adhesives, with the etch-and-rinse approach outperforming the self-etch mode. This may help explain the observed variation in Stela’s bonding performance across different etching protocols in the present study. The use of universal adhesive incorporating 10-MDP was intended to control for functional monomer chemistry, thereby enhancing comparability between the restorative systems and allowing the analysis to concentrate on restorative system–etching protocol interactions.

In contrast, the selective or short dentin etching (SDE) approach, which applies phosphoric acid for a brief duration (3 s) to dentin, leads to partial demineralization of the substrate [18]. This enhances collagen exposure, facilitating better infiltration of resin monomers and thus improving the bond strength of universal adhesives to dentin [18, 54]. The results of this study demonstrate that Stela performed better with short dentin etching compared to the self-etch mode, and although its bond strength was slightly lower than that of the etch-and-rinse protocol, the difference was not statistically significant. This suggests that extending the etching time may offer no additional bonding advantage, aligning with findings from previous studies [19]. In this study, the bond strength values for the Stela composite were lower than those reported in previous research [55], likely due to the cavity design used, which may have imposed greater stress on the bonding interface. This class-I cavity model can be considered as a worst-case scenario regarding the shrinkage stress of the overlying composite being directly imposed to the adhesive interface. Significantly lower bond strengths have been recorded in a narrow high C-factor class-I cavity mode [29, 56, 57].

In contrast, for the dual-cured composite ACTIVA, bond strength was less sensitive to the etching protocol. While the self-etch mode (19.0 ± 4.8 MPa) produced numerically lower values than short dentin etching (23.1 ± 6.8 MPa) and etch-and-rinse (26.0 ± 4.7 MPa), these differences were not statistically significant. Activa BioActive Restorative is a flowable bulk-fill restorative material that appeared in the market claiming to be an “artificial dentin” that would release calcium, phosphate, and fluoride ions to the adjacent dental tissues to reduce the possibility of recurrent caries at the margins of the cavity. The manufacturer does not specify the type of bonding agent to be used in combination with it. Thus, this study used the G-premio bond Universal. This restorative material includes a triple setting mechanism, according to the manufacturer: The acid-base neutralization reaction of GICs, light-cure, and self-cure of the matrix. This relative insensitivity to the surface pretreatment may be attributed to ACTIVA’s lower modulus of elasticity, which could allow the material to absorb and redistribute interfacial stresses, reducing the concentration of stress at the adhesive interface [58, 59]. Such stress-mitigating behaviour has been reported in other GIC-based or bioactive materials and may help preserve bond integrity, especially under challenging bonding conditions or during polymerization. The results are consistent with previous research [60], where the application of well-formulated modern adhesive systems in combination with ion-releasing dentin-replacement materials might offer clinicians the possibility to perform more long-lasting adhesive restorations.

Although the etch-and-rinse (ER) protocol with 15 s of phosphoric acid application yielded the highest mean bond strength for both materials in this study, the difference compared to the short etching approach was not statistically significant. This finding suggests that a reduced etching time may provide comparable immediate bonding performance while potentially minimizing the risks associated with aggressive demineralization. Notably, prolonged etching can increase the exposure of collagen fibrils, which if not fully infiltrated by resin, can become susceptible to enzymatic degradation by matrix metalloproteinases (MMPs) and cathepsins [52, 61, 62]. These proteolytic activities are known to compromise the hybrid layer over time, leading to interfacial breakdown and secondary caries [63]. In this context, short dentin etching may offer a clinically relevant compromise: enhancing adhesion effectiveness without introducing the long-term vulnerabilities associated with extended phosphoric acid exposure. However, the benefits of short dentin etching need to be further confirmed in long-term studies.

An earlier study [64], on primary dentin, demonstrated that reducing the etching time from 15 s to 7 s significantly influenced bond strength when a total-etch, self-priming system was used. Considering the distinct chemical, mechanical, and microstructural characteristics of primary dentin, conventional etching times recommended for permanent dentin, particularly with phosphoric acid, may be unnecessarily aggressive [64]. These findings could potentially support the concept that shortened etching protocols may be clinically advantageous and warrant reconsideration beyond primary dentin. This clinical rationale is further reinforced by another investigation [11], showing that smear layer removal with phosphoric acid significantly improved the endurance limits of resin–dentin interfaces, with the most favorable outcomes observed following shorter dentin-etching times. Although conventional 15 s etching increased fatigue strength, such benefits may be compromised in the long term by excessive collagen exposure and the subsequent risk of endogenous proteolytic degradation [11]. 

In the self-etch (SE) groups, adhesive and mixed failure modes were most common, reflecting reduced cohesive strength at the bonding interface. In contrast, the etch-and-rinse (ER) groups showed a predominance of mixed failures, indicating improved interfacial integrity and stronger bonding between the resin composite and dentin for both materials. These findings align with a previous study [55], which reported similar failure patterns. This underscores the importance of selecting an appropriate adhesive protocol, as both the restorative material and bonding strategy play a crucial role in enhancing bond durability and ensuring the long-term clinical success of restorations [52, 61]. It should be emphasized that although cavity preparation, bonding procedures, and specimen handling were standardized to maintain consistency across restorative systems, the adhesives differ in composition and application. Consequently, direct comparisons of failure modes between groups should be interpreted with caution. The reported failure patterns serve to highlight trends rather than to indicate the definitive superiority of any system.

Several limitations of this study should be acknowledged. First, as an in vitro investigation, the findings may not fully capture the complex biological and mechanical dynamics of the oral cavity, including pulpal pressure, temperature changes, enzymatic activity, and functional loading. These factors could significantly affect adhesive behavior in clinical settings. Second, the study assessed only short-term bond strength after one month of storage, which may not adequately represent the long-term durability or resistance to hydrolytic degradation of the adhesive interface. Although Class I cavities represent a clinically challenging bonding scenario, additional parameters such as marginal adaptation or interfacial gap formation were not evaluated and should be addressed in future studies to complement bond strength data. Finally, the restorative materials were applied using their respective adhesive systems in accordance with manufacturers’ instructions, reflecting current clinical practice. While this introduces material-specific variability and limits direct isolation of individual bonding mechanisms, the results provide relevant information on the overall performance of the tested restorative systems under standardized conditions. Furthermore, another limitation is the tooth dependency inherent in the microtensile bond strength (µTBS) test, as correlations between multiple specimens from the same tooth may increase the likelihood of observing statistically significant differences that do not fully reflect true group effects [65]. 

## Conclusions

Within the limitations of this in vitro study, the following conclusions can be drawn:

1. The dentin surface treatment protocol appears to affect the microtensile bond strength of the tested restorative systems in Class I cavities, with certain approaches improving early bonding performance.
2. Within the limitations of this study, the etch-and-rinse approach appears to improve immediate bond strength for both restorative systems. In addition, short dentin etching appears to improve bond strength compared with the self-etch approach, particularly for the self-cured STELA system.
3. These findings apply to the specific restorative systems and protocols evaluated and describe early bonding performance only.

## Data Availability

The data that support the findings of this study are available from the corresponding author upon reasonable request.

## Abbreviations

µTBS: Microtensile bond strength
ER: Etch-and-rinse
SE: Self-etch
SDE: Short dentin etching
C-factor: Configuration factor
RBC: Resin-based composite
RMGIC: Resin-modified glass ionomer cement
MDP (10-MDP): 10-methacryloyloxydecyl dihydrogen phosphate
HEMA: 2-hydroxyethyl methacrylate MCP – Modified calcium phosphate
MPa: Megapascal
PTF: Pre-testing failure
LED: Light-emitting diode
CEJ: Cementoenamel junction
GIC: Glass ionomer cement
MMPs: Matrix metalloproteinases
ANOVA: Analysis of variance
SD: Standard deviation

## References

[1] Buonocore MG. A simple method of increasing the adhesion of acrylic filling materials to enamel surfaces. J Dent Res.1955; 34 (6): 849–53.10.1177/00220345550340060801. 13271655 10.1177/00220345550340060801

[2] Ingles M, Vasconcelos ECJ, Mano Azul A, Polido M, Delgado AHS. Comparative assessment of different Pre-Treatment bonding strategies to improve the adhesion of Self-Adhesive composites to dentin. Polym (Basel).2022; 14 (19): 10.3390/polym14193945. 10.3390/polym14193945 PMC9570807 36235894

[3] Maciel Pires P, Davila-Sanchez A, Faus-Matoses V, Nunez Marti JM, Lo Muzio L, Sauro S. Bonding performance and ultramorphology of the resin-dentine interface of contemporary universal adhesives. Clin Oral Investig.2022; 26 (6): 4391–405.10.1007/s00784-022-04402-3. 35149904 10.1007/s00784-022-04402-3

[4] Goldberg M, Kulkarni AB, Young M, Boskey A. Dentin: structure, composition and mineralization. Front Biosci (Elite Ed).2011; 3 (2): 711–35.10.2741/e281. 21196346 10.2741/e281 PMC3360947

[5] Perdigao J, Sezinando A, Monteiro PC. Effect of substrate age and adhesive composition on dentin bonding. Oper Dent.2013; 38 (3): 267–74.10.2341/12-307-L. 23210916 10.2341/12-307-L

[6] Perdigao J. Dentin bonding-variables related to the clinical situation and the substrate treatment. Dent Mater.2010; 26 (2): e24-10.1016/j.dental.2009.11.149. 20005565 10.1016/j.dental.2009.11.149

[7] Cadenaro M, Josic U, Maravic T, Mazzitelli C, Marchesi G, Mancuso E et al. Progress in dental adhesive materials. J Dent Res.2023; 102 (3): 254–62.10.1177/00220345221145673. 36694473 10.1177/00220345221145673

[8] Sofan E, Sofan A, Palaia G, Tenore G, Romeo U, Migliau G. Classification review of dental adhesive systems: from the IV generation to the universal type. Ann Stomatol (Roma).2017; 8 (1): 1–17.10.11138/ads/2017.8.1.001. 28736601 10.11138/ads/2017.8.1.001 PMC5507161

[9] Elkaffas AA, Hamama HHH, Mahmoud SH. Do universal adhesives promote bonding to dentin? A systematic review and meta-analysis. Restor Dent Endod.2018; 43 (3): e29.10.5395/rde.2018.43.e29. 30135848 10.5395/rde.2018.43.e29 PMC6103541

[10] Hong X, Huang Z, Tong Z, Jiang H, Su M. Clinical effects of different etching modes for universal adhesives: a systematic review and meta-analysis. Ann Palliat Med.2021; 10 (5): 5462–7310.21037/apm-21-890. 34107709 10.21037/apm-21-890

[11] Stape THS, Viita-Aho T, Sezinando A, Wik P, Mutluay M, Tezvergil-Mutluay A. To etch or not to etch, part I: on the fatigue strength and dentin bonding performance of universal adhesives. Dent Mater.2021; 37 (6): 949–60.10.1016/j.dental.2021.02.016. 33838928 10.1016/j.dental.2021.02.016

[12] Perdigao J. Current perspectives on dental adhesion: (1) dentin adhesion - not there yet. Jpn Dent Sci Rev.2020; 56 (1): 190–207.10.1016/j.jdsr.2020.08.004. 34188727 10.1016/j.jdsr.2020.08.004 PMC8216299

[13] Perdigao J, Ceballos L, Giraldez I, Baracco B, Fuentes MV. Effect of a hydrophobic bonding resin on the 36-month performance of a universal adhesive-a randomized clinical trial. Clin Oral Investig.2020; 24 (2): 765–76.10.1007/s00784-019-02940-x. 31147827 10.1007/s00784-019-02940-x

[14] Josic U, Maravic T, Mazzitelli C, Radovic I, Jacimovic J, Del Bianco F et al. Is clinical behavior of composite restorations placed in non-carious cervical lesions influenced by the application mode of universal adhesives? A systematic review and meta-analysis. Dent Mater.2021; 37 (11): e503–e10.1016/j.dental.2021.08.017. 34481667 10.1016/j.dental.2021.08.017

[15] Zhang ZY, Tian FC, Niu LN, Ochala K, Chen C, Fu BP et al. Defying ageing: an expectation for dentine bonding with universal adhesives? J Dent.2016; 45 43–52.10.1016/j.jdent.2015.11.008. 26655173 10.1016/j.jdent.2015.11.008

[16] Atalay C, Ozgunaltay G, Yazici AR. Thirty-six-month clinical evaluation of different adhesive strategies of a universal adhesive. Clin Oral Investig.2020; 24 (4): 1569–78.10.1007/s00784-019-03052-2. 31468262 10.1007/s00784-019-03052-2

[17] de Paris Matos T, Perdigao J, de Paula E, Coppla F, Hass V, Scheffer RF et al. Five-year clinical evaluation of a universal adhesive: A randomized double-blind trial. Dent Mater.2020; 36 (11): 1474–85.10.1016/j.dental.2020.08.007. 32933775 10.1016/j.dental.2020.08.007

[18] Stape THS, Wik P, Mutluay MM, Al-Ani AAS, Tezvergil-Mutluay A. Selective dentin etching: A potential method to improve bonding effectiveness of universal adhesives. J Mech Behav Biomed Mater.2018; 86 14–22.10.1016/j.jmbbm.2018.06.015. 29913306 10.1016/j.jmbbm.2018.06.015

[19] Ismail HS, Soliman HAN. Short dentin etching with universal adhesives: effect on bond strength and gingival margin adaptation. BMC Oral Health.2025; 25 (1): 128.10.1186/s12903-025-05490-9. 39849440 10.1186/s12903-025-05490-9 PMC11760696

[20] Hardan L, Orsini G, Bourgi R, Cuevas-Suarez CE, Nicastro M, Lazarescu F et al. Effect of active bonding application after selective dentin etching on the immediate and Long-Term bond strength of two universal adhesives to dentin. Polym (Basel).2022; 14 (6): 10.3390/polym14061129. 10.3390/polym14061129 PMC8951477 35335459

[21] Leprince JG, Palin WM, Vanacker J, Sabbagh J, Devaux J, Leloup G. Physico-mechanical characteristics of commercially available bulk-fill composites. J Dent.2014; 42 (8): 993–1000.10.1016/j.jdent.2014.05.009. 24874951 10.1016/j.jdent.2014.05.009

[22] Van Ende A, De Munck J, Lise DP, Van Meerbeek B. Bulk-Fill composites: A review of the current literature. J Adhes Dent.2017; 19 (2): 95–109.10.3290/j.jad.a38141. 28443833 10.3290/j.jad.a38141

[23] Ghavami-Lahiji M, Hooshmand T. Analytical methods for the measurement of polymerization kinetics and stresses of dental resin-based composites: A review. Dent Res J (Isfahan).2017; 14 (4): 225–40.10.4103/1735-3327.211628. 28928776 10.4103/1735-3327.211628 PMC5553250

[24] Betancourt DE, Baldion PA, Castellanos JE. Resin-Dentin bonding interface: mechanisms of degradation and strategies for stabilization of the hybrid layer. Int J Biomater. 2019;2019:5268342. 10.1155/2019/5268342. 30853990 10.1155/2019/5268342 PMC6378048

[25] Tjan AH, Bergh BH, Lidner C. Effect of various incremental techniques on the marginal adaptation of class II composite resin restorations. J Prosthet Dent.1992; 67 (1): 62–6.10.1016/0022-3913(92)90051-b. 1548611 10.1016/0022-3913(92)90051-b

[26] Thadathil Varghese J, Raju R, Farrar P, Prentice L, Prusty BG. Comparative analysis of self-cure and dual cure-dental composites on their physico-mechanical behaviour. Aust Dent J.2024; 69 (2): 124–38.10.1111/adj.13004. 38131257 10.1111/adj.13004

[27] Pires PM, de Almeida Neves A, Lukomska-Szymanska M, Farrar P, Cascales AF, Sauro S. Bonding performance and interfacial adaptation of modern bulk-fill restorative composites after aging in artificial saliva: an in vitro study. Clin Oral Investig.2024; 28 (2): 132.10.1007/s00784-024-05525-5. 38308668 10.1007/s00784-024-05525-5

[28] Yao C, Ahmed MH, Okazaki Y, Van Landuyt KL, Huang C, Van Meerbeek BB. Efficacy of a new Self-Adhesive restorative onto flat dentin vs Class-I Cavity-bottom dentin. J Adhes Dent.2020; 22 (1): 65–77.10.3290/j.jad.a43999. 32030377 10.3290/j.jad.a43999

[29] Yao C, Ahmed MH, Zhang F, Mercelis B, Van Landuyt KL, Huang C et al. Structural/Chemical characterization and bond strength of a new Self-Adhesive Bulk-fill restorative. J Adhes Dent.2020; 22 (1): 85–97.10.3290/j.jad.a44000. 32030379 10.3290/j.jad.a44000

[30] Lardani L, Derchi G, Marchio V, Carli E. One-Year clinical performance of activa Bioactive-Restorative composite in primary molars. Child (Basel).2022; 9 (3): 10.3390/children9030433. 10.3390/children9030433 PMC8946891 35327805

[31] Abdel-Maksoud HB, Bahanan AW, Alkhattabi LJ, Bakhsh TA. Evaluation of newly introduced bioactive materials in terms of cavity floor adaptation: OCT study. Mater (Basel).2021; 14 (24): 10.3390/ma14247668. 10.3390/ma14247668 PMC8708320 34947264

[32] Popa M, Dinu S, Luca MM, Bumbu BA, Maghet E, Bita RG. Clinical and laboratory performance of ACTIVA bioactive restorative in primary teeth: A systematic review of pediatric evidence. J Clin Med.2026; 15 (1): 10.3390/jcm15010373. 10.3390/jcm15010373 PMC12786899 41517620

[33] van Dijken JWV, Pallesen U. Benetti A.A randomized controlled evaluation of posterior resin restorations of an altered resin modified glass-ionomer cement with claimed bioactivity. Dent Mater.2019; 35 (2): 335–43.10.1016/j.dental.2018.11.027. 30527586 10.1016/j.dental.2018.11.027

[34] Pinto NS, Jorge GR, Vasconcelos J, Probst LF, De-Carli AD, Freire A. Clinical efficacy of bioactive restorative materials in controlling secondary caries: a systematic review and network meta-analysis. BMC Oral Health.2023; 23 (1): 394.10.1186/s12903-023-03110-y. 37322456 10.1186/s12903-023-03110-y PMC10268411

[35] de Carvalho LF, Gimenes ESM, Barboza ADS, Badaro MM, Stolf SC, Cuevas-Suarez CE et al. Effectiveness of bioactive resin materials in preventing secondary caries and retention loss in direct posterior restorations: A systematic review and meta-analysis. J Dent.2025; 152105460.10.1016/j.jdent.2024.105460. 10.1016/j.jdent.2024.105460 39547467

[36] Al-Harbi F, Kaisarly D, Bader D, El Gezawi MM. Integrity of bulk versus incremental fill class II composite restorations. Oper Dent.2016; 41 (2): 146–56.10.2341/14-306-L. 26266653 10.2341/14-306-L

[37] Kim RJ, Kim YJ, Choi NS, Lee IB. Polymerization shrinkage, modulus, and shrinkage stress related to tooth-restoration interfacial debonding in bulk-fill composites. J Dent.2015; 43 (4): 430–910.1016/j.jdent.2015.02.002. 25676178 10.1016/j.jdent.2015.02.002

[38] Van Meerbeek B, Peumans M, Poitevin A, Mine A, Van Ende A, Neves A et al. Relationship between bond-strength tests and clinical outcomes. Dent Mater.2010; 26 (2): e100-10.1016/j.dental.2009.11.148. 20006379 10.1016/j.dental.2009.11.148

[39] Betamar N, Cardew G, Van Noort. R.Influence of specimen designs on the microtensile bond strength to dentin. J Adhes Dent.2007; 9 (2): 159–68.https://doi.org/. 17489476

[40] Pashley DH, Carvalho RM, Sano H, Nakajima M, Yoshiyama M, Shono Y et al. The microtensile bond test: a review. *J Adhes Dent.*1999; 1 (4): 299–309.https://doi 11725659

[41] Nikaido T, Kunzelmann KH, Ogata M, Harada N, Yamaguchi S, Cox CF, et al. The in vitro dentin bond strengths of two adhesive systems in class I cavities of human molars. J Adhes Dent. 2002;4(1):31–9. https://doi 12071627

[42] Armstrong SR, Keller JC, Boyer DB. The influence of water storage and C-factor on the dentin-resin composite microtensile bond strength and debond pathway utilizing a filled and unfilled adhesive resin. Dent Mater.2001; 17 (3): 268–76.10.1016/s0109-5641(00)00081-6. 11257301 10.1016/s0109-5641(00)00081-6

[43] Nikaido T, Kunzelmann KH, Chen H, Ogata M, Harada N, Yamaguchi S et al. Evaluation of thermal cycling and mechanical loading on bond strength of a self-etching primer system to dentin. Dent Mater.2002; 18 (3): 269–75.10.1016/s0109-5641(01)00048-3. 11823020 10.1016/s0109-5641(01)00048-3

[44] Armstrong S, Breschi L, Ozcan M, Pfefferkorn F, Ferrari M, Van Meerbeek B. .Academy of dental materials guidance on in vitro testing of dental composite bonding effectiveness to dentin/enamel using micro-tensile bond strength (muTBS) approach. Dent Mater.2017; 33 (2): 133–43.10.1016/j.dental.2016.11.015. 28007396 10.1016/j.dental.2016.11.015

[45] Choi AN, Lee JH, Son SA, Jung KH, Kwon YH, Park JK. Effect of dentin wetness on the bond strength of universal adhesives. Mater (Basel).2017; 10 (11): 10.3390/ma10111224. 10.3390/ma10111224 PMC5706171 29068404

[46] Murray PE, Smith AJ, Windsor LJ, Mjor IA. Remaining dentine thickness and human pulp responses. Int Endod J.2003; 36 (1): 33–43.10.1046/j.0143-2885.2003.00609.x. 12656512 10.1046/j.0143-2885.2003.00609.x

[47] Kumagai RY, Hirata R, Pereira PNR, Reis AF. Moist vs over-dried etched dentin: FE-SEM/TEM and bond strength evaluation of resin-dentin interfaces produced by universal adhesives. J Esthet Restor Dent.2020; 32 (3): 325–32.10.1111/jerd.12537. 31622014 10.1111/jerd.12537

[48] Yoshikawa T, Sadr A, Tagami J. Effects of C-factor on bond strength to floor and wall dentin. Dent Mater J.2016; 35 (6): 918–22.10.4012/dmj.2016-111. 27725368 10.4012/dmj.2016-111

[49] Rosa WL, Piva E, Silva AF. Bond strength of universal adhesives: A systematic review and meta-analysis. J Dent.2015; 43 (7): 765–76.10.1016/j.jdent.2015.04.003. 25882585 10.1016/j.jdent.2015.04.003

[50] Fehrenbach J, Isolan CP, Munchow EA. Is the presence of 10-MDP associated to higher bonding performance for self-etching adhesive systems? A meta-analysis of in vitro studies. Dent Mater.2021; 37 (10): 1463–8510.1016/j.dental.2021.08.014. 34456050 10.1016/j.dental.2021.08.014

[51] Cardoso GC, Nakanishi L, Isolan CP, Jardim PDS, Moraes RR. Bond stability of universal adhesives applied to dentin using Etch-And-Rinse or Self-Etch strategies. Braz Dent J.2019; 30 (5): 467–75.10.1590/0103-6440201902578. 31596331 10.1590/0103-6440201902578

[52] Fan-Chiang YS, Chou PC, Hsiao YW, Cheng YH, Huang Y, Chiu YC et al. Optimizing dental bond strength: insights from comprehensive literature review and future implications for clinical practice. Biomedicines.2023; 11 (11): 10.3390/biomedicines11112995. 10.3390/biomedicines11112995 PMC10669570 38001996

[53] Tay FR, Pashley DH. Aggressiveness of contemporary self-etching systems. I: depth of penetration beyond dentin smear layers. Dent Mater.2001; 17 (4): 296–308.10.1016/s0109-5641(00)00087-7. 11356206 10.1016/s0109-5641(00)00087-7

[54] Kharouf N, Rapp G, Mancino D, Hemmerle J, Haikel Y, Reitzer F. Effect of etching the coronal dentin with the rubbing technique on the microtensile bond strength of a universal adhesive system. Dent Med Probl.2019; 56 (4): 343–810.17219/dmp/111697. 31794165 10.17219/dmp/111697

[55] Pires PM, Almeida Neves A, Farrar P, Ferrando Cascales A, Banerjee A, Pinheiro Feitosa V et al. Bonding performance and interfacial Ultra-Morphology/Nanoleakage of a modern Self-Curing Bulk-Fill restorative system: an in vitro study. Eur J Dent.2025; 10.1055/s-0045-1804886. 40132978 10.1055/s-0045-1804886 PMC12890399

[56] Van Ende A, De Munck J, Van Landuyt K, Van Meerbeek B. Effect of Bulk-filling on the bonding efficacy in occlusal class I cavities. J Adhes Dent.2016; 18 (2): 119–24.10.3290/j.jad.a35905. 27042703 10.3290/j.jad.a35905

[57] Van Ende A, De Munck J, Van Landuyt KL, Poitevin A, Peumans M, Van Meerbeek B. Bulk-filling of high C-factor posterior cavities: effect on adhesion to cavity-bottom dentin. Dent Mater.2013; 29 (3): 269–77.10.1016/j.dental.2012.11.002. 23228335 10.1016/j.dental.2012.11.002

[58] Nikolaenko SA, Lohbauer U, Roggendorf M, Petschelt A, Dasch W, Frankenberger R. Influence of c-factor and layering technique on microtensile bond strength to dentin. Dent Mater.2004; 20 (6): 579–85.10.1016/j.dental.2003.08.001. 15134946 10.1016/j.dental.2003.08.001

[59] Irie M, Suzuki K, Watts DC. Immediate performance of self-etching versus system adhesives with multiple light-activated restoratives. Dent Mater.2004; 20 (9): 873–80.10.1016/j.dental.2004.04.003. 15451243 10.1016/j.dental.2004.04.003

[60] Sauro S, Makeeva I, Faus-Matoses V, Foschi F, Giovarruscio M, Maciel Pires P et al. Effects of Ions-Releasing restorative materials on the dentine bonding longevity of modern universal adhesives after Load-Cycle and prolonged artificial saliva aging. Mater (Basel).2019; 12 (5): 10.3390/ma12050722. 10.3390/ma12050722 PMC6427106 30832247

[61] Carvalho RM, Manso AP, Geraldeli S, Tay FR, Pashley DH. Durability of bonds and clinical success of adhesive restorations. Dent Mater.2012; 28 (1): 72–86.10.1016/j.dental.2011.09.011. 22192252 10.1016/j.dental.2011.09.011 PMC3863938

[62] Sebold M, Giannini M, Andre CB, Sahadi BO, Maravic T, Josic U et al. Bonding interface and dentin enzymatic activity of two universal adhesives applied following different etching approaches. Dent Mater.2022; 38 (6): 907–23.10.1016/j.dental.2022.03.001. 35289283 10.1016/j.dental.2022.03.001

[63] Josic U, D’Alessandro C, Miletic V, Maravic T, Mazzitelli C, Jacimovic J et al. Clinical longevity of direct and indirect posterior resin composite restorations: an updated systematic review and meta-analysis. Dent Mater.2023; 39 (12): 1085–9410.1016/j.dental.2023.10.009. 37827872 10.1016/j.dental.2023.10.009

[64] Sardella TN, de Castro FL, Sanabe ME, Hebling J. Shortening of primary dentin etching time and its implication on bond strength. J Dent.2005; 33 (5): 355–62.10.1016/j.jdent.2004.10.011. 15833390 10.1016/j.jdent.2004.10.011

[65] Hannigan A, Lynch CD. Statistical methodology in oral and dental research: pitfalls and recommendations. J Dent.2013; 41 (5): 385–92.10.1016/j.jdent.2013.02.013. 23459191 10.1016/j.jdent.2013.02.013

